# Hemodynamic oxygenator exchange-related effects during veno-venous extracorporeal membrane oxygenation for the treatment of acute SARS-CoV-2 respiratory distress syndrome

**DOI:** 10.1177/02676591211056564

**Published:** 2022-03-04

**Authors:** Sébastien Colombier, Adrien Gross, Antoine Schneider, Piergiorgio Tozzi, Zied Ltaief, Mario Verdugo-Marchese, Matthias Kirsch, Lars Niclauss

**Affiliations:** 1 Department of Cardiovascular Surgery, 30635 Lausanne University Hospital (CHUV), Switzerland; 2 Department of Anaesthesiology, 30635 Lausanne University Hospital (CHUV), Switzerland; 3 Department of Intensive Care Unit, 30635 Lausanne University Hospital (CHUV), Switzerland

**Keywords:** ARDS, veno-venous extracorporeal membrane oxygenation, membrane oxygenator replacement, ECMO circuit, hypoxemia, COVID-19

## Abstract

Few patients with coronavirus disease 2019–associated severe acute respiratory distress syndrome (ARDS) require veno-venous extracorporeal membrane oxygenation (VV-ECMO). Prolonged VV-ECMO support necessitates repeated oxygenator replacement, increasing the risk for complications. Transient hypoxemia, induced by VV-ECMO stop needed for this procedure, may induce transient myocardial ischemia and acutely declining cardiac output in critically ill patients without residual pulmonary function. This is amplified by additional activation of the sympathetic nervous system (tachycardia, pulmonary vasoconstriction, and increased systemic vascular resistance). Immediate reinjection of the priming solution of the new circuit and induced acute iatrogenic anemia are other potentially reinforcing factors. The case of a critically ill patient presented here provides an instructive illustration of the hemodynamic relationships occurring during VV-ECMO support membrane oxygenator exchange.

## Introduction

The coronavirus disease (COVID-19) outbreak is characterized by a high rate of hospitalized patients with respiratory failure. Twenty percent of them develop an acute respiratory distress syndrome (ARDS) requiring ventilation.^1^ According to Extracorporeal Life Support Organization recommendations and World Health Organization interim guidelines, approximately 1%–5% of these ventilated patients will benefit from veno-venous extracorporeal membrane oxygenation (VV-ECMO) as a last therapeutic option.^2-4^ Initial clinical experience suggests that these patients can be successfully treated; however, this invasive therapy also carries an increased risk of complications. This is particularly due to the inflammatory and pro-thrombotic state, with increased coagulation activities and hemolysis observed.^5-7^ Accordingly, prolonged VV-ECMO support requires repeated membrane oxygenator exchange. This is generally a straightforward procedure; however, in critically ill patients without residual pulmonary function, significant transient hemodynamic impairment may occur.

## Case history

A 60-year-old previously healthy patient was admitted for ARDS caused by SARS-CoV-2. The patient required oro-tracheal intubation and mechanical ventilation. 9 days later, he became refractory to conventional therapies. Despite prone positioning, neuromuscular blockade, and high positive end-expiratory pressure ventilation, the ratio of partial pressure of oxygen in arterial blood to fractional concentration of oxygen in inspired air (PaO_2_/FiO_2_) was below 80 mmHg for more than 6 h. A VV-ECMO was implanted. After heparinisation (intra-venous injection of 5000 UI unfractionated heparin), a 25 French (Fr) 55-cm inflow multiport cannula (Maquet®) was inserted percutaneously via the right femoral vein and a 17 Fr 23-cm outflow simple-port cannula (Maquet®) via the right internal jugular vein. The cannulas were connected to an HLS circuit (Bioline Coating, Maquet®) and a Cardiohelp® device. VV-ECMO support was initiated without problems, and systematic heparinisation was monitored by measuring anti-Xa factor activity (0.3–0.5 units/mL).

**Figure 1.**
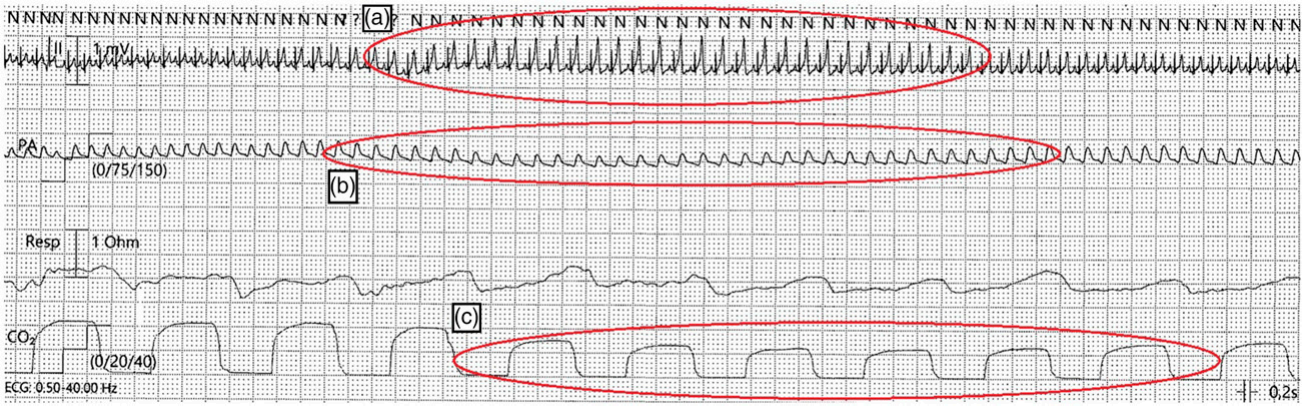
Monitoring immediately after membrane oxygenator and circuit exchange. (a) Electrocardiogram showing tachycardia, large peaked T waves and ST segment elevation (red circle). (b) Simultaneous blood pressure drop of the continuous invasive radial measurement (red circle). (c) Drop in continuous end-tidal CO2 monitoring (red circle).

8 days later, the oxygenator had to be replaced for the first time because of severe hemolysis (haptoglobin was less than 0.1 g/L, with a normal range of 0.3–2.0 g/L). This was realized without complications, and hemolysis regressed. However, the membrane oxygenator had to be replaced again on days 16, 24, and 36 because of the same problem. The ECMO circuit was disconnected during this procedure and replaced by a new one (HLS Maquet®) previously primed with 600 mL of isotonic crystalloids (Ringer’s lactate, Fresenius®). During one of these changes, after reconnection of the previously primed circuit as well as VV-ECMO restart, electrocardiographic (ECG) monitoring showed large, peaked T waves as well as ST segment elevation (Figure 1(a)). Few seconds later, there was a decrease in systemic arterial blood pressure and a decrease in end-tidal (end-expiratory) carbon dioxide during continuous monitoring (Figure 1(b) and (c)). These changes persisted for approximately 1 min before all corresponding parameters normalized (Figure 1(a)–(c)).

The circuit change was performed rapidly (29 s) and was otherwise technically unproblematic. Pre-procedurally, hemoglobin was 112 g/L (normal range 133–177 g/L) and then 98 g/L, corresponding to a 15% decrease. High sensitive troponin T did not increase, and echocardiography showed unchanged normal left ventricular ejection fraction. After further stabilization, the patient could be weaned off VV-ECMO after 45 days. The patient left the intensive care unit on day 85.

## Discussion

Long-term VV-ECMO patients are known to undergo a number of acute biological changes, including alterations in coagulation activity. In addition, approximately one-third of critically ill COVID-19 patients develop coagulopathy, with thrombotic events predominating over haemorrhagic events.^5^ Therefore, it seems not surprising that accordingly, in COVID-19 patients under VV-ECMO support, repeated oxygenator changes become necessary.

The hemodynamic changes observed in this case are most likely triggered by the short-term severe acute hypoxemia caused by the sudden cessation of VV-ECMO support in patients with severely impaired residual pulmonary function. The transient decrease in oxygen delivery leads to depressed myocardial function, wall motion abnormalities, arrhythmias, and corresponding ECG changes similar to cardiac ischemia.^8^ In the early hypoxemic phase of our patient, the ECG shows large, peaked (hyper-acute) T waves followed by the appearance of ST elevations, indicating acute myocardial ischemia (Figure 1(a)). These changes could indicate the possible presence of coronary artery disease, which would explain the lack of a corresponding physiological compensatory increase in coronary blood flow, as a normal response to hypoxemia.^8,9^ In addition, activation of the sympathetic nervous system increases heart rate, pulmonary circulation afterload (vasoconstriction), and systemic vascular resistance, resulting in additional stress on the already weakened myocardium.^8,9^

Therefore, as a consequence of this transient hypoxemia and subsequent cardiac ischemia, there may be a decrease in cardiac output, systemic hypotension, and a decrease in end-tidal CO_2_ (Figure 1(b) and (c)).

Although these mechanisms are effective for a very short time, they are exacerbated by the connection of the new circuit and the subsequent immediate reinjection of the crystalloid priming solution because this leads to a rapid decrease in hematocrit and hemoglobin concentration that immediately follows the hypoxemia provoked by the VV-ECMO stop. This iatrogenic anemia could therefore be another factor exacerbating hypoxia-induced myocardial weakness.^8^ Restarting VV-ECMO at a reduced flow rate could potentially attenuate this “crystalloid flush effect.”

## Conclusion

The hemodynamic changes shown here confirm that membrane oxygenator replacement carries some risks, especially in patients with severe respiratory failure. This knowledge can help to follow certain safety rules and to react adequately.
